# Clinical Observation and Genotype-Phenotype Analysis of ABCA4-Related Hereditary Retinal Degeneration before Gene Therapy

**DOI:** 10.2174/1566523222666220216101539

**Published:** 2022-06-01

**Authors:** Xuan Xiao, Lin Ye, Changzheng Chen, Hongmei Zheng, Jiajia Yuan

**Affiliations:** 1 Eye Center, Renmin Hospital of Wuhan University, Wuhan University, Hubei, Wuhan 430060, China;; 2 Department of Eye Plastic and Lacrimal Diseases, Shenzhen Eye Hospital, Shenzhen, China

**Keywords:** *ABCA4*, stargardt disease, cone cell malnutrition, retinitis pigmentosa (cone type), cone-rod dystrophy, retinal degeneration

## Abstract

**Background:**

Hereditary retinal degeneration (HRD) is an irreversible eye disease that results in blindness in severe cases. It is most commonly caused by variants in the *ABCA4* gene. HRD presents a high degree of clinical and genetic heterogeneity. We determined genotypic and phenotypic correlations, in the natural course of clinical observation, of unrelated progenitors of HRD associated with *ABCA4*.

**Objective:**

To analyze the relationship between the phenotypes and genotypes of *ABCA4* variants.

**Methods:**

A retrospective clinical study of five cases from the ophthalmology department of the People’s Hospital of Wuhan University from January 2019 to October 2020 was conducted. We tested for *ABCA4* variants in the probands. We performed eye tests, including the best-corrected visual acuity, super-wide fundus photography and spontaneous fluorescence photography, optical coherence tomography, and electrophysiological examination.

**Results:**

Disease-causing variants were identified in the *ABCA4* genes of all patients. Among these, seven *ABCA4* variants were novel. All patients were sporadic cases; only one patient had parents who were relatives, and the other four patients were offspring of unrelated parents. Two patients presented with Stargardt disease, mainly with macular lesions, two presented with retinitis pigmentosa (cone-rod type), and one presented with cone dystrophy. The visual acuity and visual field of the five patients showed varying degrees of deterioration and impairment.

**Conclusion:**

The same *ABCA4* mutation can lead to different clinical phenotypes, and there is variation in the degree of damage to vision, visual field, and electrophysiology among different clinical phenotypes. Clinicians must differentiate between and diagnose pathologies resulting from this mutation.

## 1 INTRODUCTION

Hereditary retinal degeneration (HRD) is a serious, irreversible eye disease causing visual impairment, resulting in blindness in severe cases. The disease is mainly caused by mutation and has a high degree of clinical and genetic heterogeneity. Mutation on the *ABCA4* gene is the most common cause of HRD [1, 2]. Hundreds of *ABCA4* variants have been reported to date. Therefore, the corresponding clinical phenotypes are also complex and variable. Possible clinical manifestations include Stargardt disease (STGD) [3-6], recessive inherited retinitis pigmentosa (RP) [7-9], cone dystrophy (COD), and cone-rod dystrophy (CORD) [9, 10]. *ABCA4* gene variants have an incidence of 4.5%~10% in the general populace. It is located on 1p221, contains 50 exons,and is responsible for coding the RmP protein. RmP has a molecular mass of 256 kDa and is mainly localized in the outer membrane segment of retinal photoreceptor cells. It plays a key role in the retinal photochemical cycle by removing all-trans-retinal and phosphatidyl ethanolamine reaction products of N–retinylidene–N retinyl ethanolamine, A2E [11-14].

However, variants of *ABCA4* can lead to a dysfunctional RmP protein, causing A2E to accumulate in the retinal pigment epithelium, resulting in damage to its structure and function, and finally leading to the development of RP [15, 16]. STGD is common macular dystrophy and hereditary retinal disease, primarily manifesting as progressive central vision loss, and its prevalence is approximately 1 in 10,000. STGD usually begins its onset in adolescents [17], and mutation of the *ABCA4* gene is the most common causative mutation of STGD. Besides progressive deterioration of central vision, the typical positive signs are round or oval atrophy foci in the macular area of the fundus and yellow-white spots in the surrounding retina.

RP is a serious and progressive eye disease, with an incidence of 1 in 3,500 to 1 in 4,000 [7-9, 18]. One subtype is rod-cone RP, in which rod cells show decreased function at early stages, and at the later stage, the cone cells also exhibit decreased function. The main clinical manifestations are early night blindness and central vision loss. The typical changes within the fundus are the waxy yellow appearance of the optic papillae, narrowing of the retinal artery, and characteristic osteocellular pigment deposition in the periphery. At present, there are at least 50 known RP-related pathogenic genes, among which the RP (rod-cone type) caused by *ABCA4* mutation often shows a severe clinical phenotype.

COD is a form of hereditary macular degeneration that mainly damages cone cells. The main clinical manifestations are the disappearance of light reflection in the macular fovea, the bluish-gray appearance of the macular area with gold-foil-like reflection, and atrophy of the retinal pigment epithelium.

In this study, five *ABCA4* patients were examined in the laboratory and followed up clinically. We examined the relationship between their genotypes and phenotypes in terms of morphology and function to provide a valuable reference for future clinical diagnosis.

## 2 MATERIALS AND METHODS

### 2.1 General Condition of Patients

We selected five patients with *ABCA4* gene variants who visited the ophthalmology department of the People’s Hospital of Wuhan University from January 2019 to October 2020 as the research subjects. We collected basic information from all the patients and tested their peripheral blood samples for exons of the *ABCA4* gene. Written informed consent was collected from all the patients involved in this study.

### 2.2 Genetic Testing

The dual-terminal sequencing method was mainly based on an Illumina HiSeqX platform (San Diego, CA, USA). The total data obtained by sequencing were 10.69 Gb, and over 85% of bases had a sequencing quality score ^3^ Q30. Burrows-Wheeler Aligner(BWA v0.7.16a) was used to align reads to human assembly hg19(GRCh37). The average sequencing depth of the exome sequencing target region was 105.57×, and the coverage was over 99.25%, in which the sequencing depth of the target sequence was 96.0% and above 20×. Then, ANNOVAR (http://annovar.openbioinformatics.org/) software was used to perform mutation site point annotations and obtain candidate pathogenic mutation sites. Finally, the analysis and clinical significance were screened for pathogenic variation according to the ACMG variation classification guidelines and the clinical information of the subjects. We used the generation Sanger platform to detect and verify sites of variation that could explain the clinical phenotypes of the subjects.

### 2.3 Eye Examinations

We performed ophthalmic examinations, including best-corrected visual acuity, ultra-wide-angle fundus, autofluorescence, the field of vision, optical coherence tomography, electroretinography (ffERG), and multifocal electroretinography (mfERG), on all five proband patients.

The same ophthalmologist conducted visual acuity tests based on the best-corrected visual acuity. Eye charts were 2.5 m standard logMAR charts (Star Kang Medical Technology Co. Ltd, Wenzhou, China), in which one line represented 0.3 log MAR units. The ophthalmologist examined patients three times at 3 min intervals to confirm changes in visual acuity, and we considered the mean value to be the final visual acuity.

The tonometer used for determining intraocular pressure (IOP) was a TOPCON-CT-80 Computerized Auto Tonometer (Topcon, Tokyo, Japan). We used the mean value of three measurements.

For fundus (retinal) photography, we used the NIDEK Autofocus Fundus Camera, AFC-230 (Nidek, Tokyo, Japan). We conducted visual field tests using a Humphrey field analyzer (740i, Carl Zeiss, Shanghai, China). The testing procedure included the 30–2 central threshold test and SITA fast. The major recorded parameters were visual field index (VFI) and mean defect (MD).

For visual evoked potential (VEP), the function of the optic nerve was examined after the value of the P100 wave was determined using COLORDOME (Diagnosys LLC, Lowell, MA, USA). We optically corrected tested eyes and adopted a binocular viewing condition. We acquired and analyzed the data with a connected computer.

FfERG and mfERG were also performed using ColorDome (Diagnosys LLC). Optical coherence tomography (OCT) was performed with a Spectralis^®^ HRA+OCT (Heidelberg Engineering, Heidelberg, Germany), and retinal nerve fiber layer (RNFL) thickness measurements of the four quadrants (bottom, top, left, and right quadrants) were analyzed. All OCT data were automatically calculated using Spectralis^®^ HRA+OCT software. All examinations were conducted by the same technicians within the Ophthalmology Department of the People’s Hospital of Wuhan University (Wuhan, China).

## 3 RESULTS

### 3.1 Patients

All five patients were diagnosed as carrying a mutant *ABCA4* gene. Among the five patients, three were males, and two were females, with an average age of 20.4 ± 8.79 (10–33) years old. The parents of Patient no. 1 were related, while the other four patients were the offspring of normal marriages. The clinical course of the five patients was 8.6 ± 5.89 (2–16) years. No patients reported any obvious family history (Table **1**).

### 3.2 Mutation Identification

**Fig. (1):**
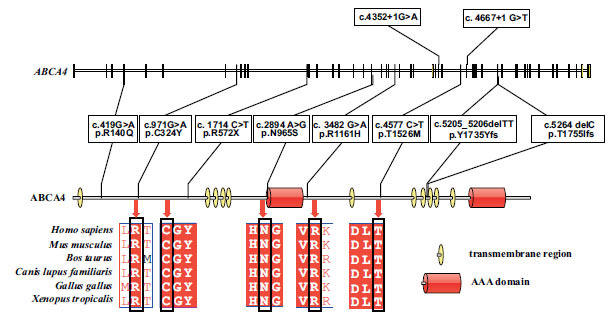
*ABAC4* gene structure and conserved analysis. Conservation analysis of five missense mutations on ABCA4 by multiple sequence alignment (ClustalW). Organisms aligned include *Homo sapiens*, *Mus musculus*, *Bos taurus*, *Canis lupus familiaris*, *Gallus gallus,* and *Xenopus tropicalis*. The conserved analysis finds that those five missense mutation sites are conserved in the most common species, indicating that variants in those sites may have higher pathogenicity.

To identify the molecular basis of HRD in all patients, we performed whole-exome sequencing in all five patients. In our patient cohort, we identified 10 mutations in ABCA4's coding region and intronic regions. Eight of them were missense variants, and two of them were deletion variants. A Pure variant p.T1755Ifs was observed in patient 1 whose parents were related, but we did not get his parents’ sequencing information. In patient 2, we found Compound heterozygous variants; p.Y1735Yfs variant was heterozygous in his father but absent in the mother, while the variant p.C324Y was heterozygous in his mother but not detected in his father. Patient 3 had two heterozygous variants from the mother, c.4667+1G > T and p.R140Q. At the same time, patient 3 had a splicing mutation, c.4352+1G > A, which we could not determine the source of since we did not have the father's information. For patients 4, p.N965S and p.T1526M were observed; one came from his father, another was detected in his mother. Two-point variants were detected in patient 5, p.R572X heterozygous in her father, while p.R1161H heterozygous in her mother (Fig. **1**).

### 3.3 Visual Acuity and Fundus Photography

**Fig. (2):**
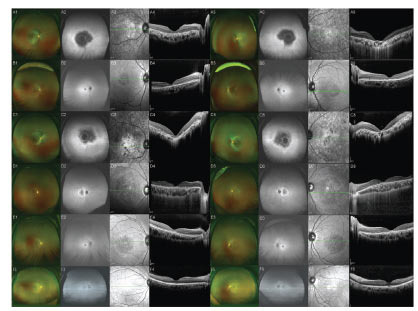
We performed fundus photography, spontaneous fluorescence fundus photography, and macular OCT examination in five patients. No. 1: A1-A4 (right eye), A5-A8 (left eye); No. 2: B1-B4 (right eye), B5-B8 (left eye); No. 3: C1-C4 (right eye), C5-C8 (left eye); No. 4: D1-D4 (right eye), D5-D8 (left eye); No. 5: E1-E4 (right eye), E5-E8 (left eye); No. 6: F1-F4 (right eye), F5-F8 (left eye). Patients no. 1 and 3 presented with a total retinal lesion with osteoid cell deposition in the posterior pole and peripheral retina, with thinning of the macular area. Patients no. 2 and 4 mainly presented with a transverse elliptic zone of clear atrophy in the macular area and small grayish-yellow spots in the deep macular area. Patient no. 5 presented with macular area cone damage without significant thinning. Patient 6 was a healthy control. (A higher resolution / colour version of this figure is available in the electronic copy of the article).

The visual acuity of the five patients was 1.28 ± 0.26 and 1.3 ± 0.37 LogMAR in the right and left eye, respectively. IOP was 14.56 ± 2.22 mmHg in the right eye and 15.24 ± 2.02 mmHg in the left eye (Table **2**). The retinal osteocellular pigmentation in Patients no. 1 and 3 involved the equator, and the optic disc was yellowish. OCT showed that the structure of the macular area was thinner, the structure of the RPE layer was disordered, and there was the deposition of some metabolic substance. The structure of the peripheral retina’s inner layer was mostly intact. OCT in Patient no. 3 showed depression of the choroid in the macular area. Patients no. 2 and no. 4 showed no central reflection in the fundus, small grayish-yellow spots in the deep macula, and the gradual formation of a shrinking area with a clear transverse elliptic boundary, with a transverse diameter of about 1.5–2 PD and a diameter of approximately 1.0–1.5 PD. OCT showed atrophy and thinning in the macular area of both eyes, showing an early stage of disease profession (Fig. **2**).

### 3.4 Visual Field and RNFL

The visual field of the five patients showed varying degrees of defects. The average visual field indices of the right and left eye were 50. 6 ± 27.94% and 52.2 ± 29.43%, respectively. The average defect values of the right and left eyes were -16.59 ± 10.25 vdB and -15.93 ± 10.45 vdB, respectively. The thickness of the optic nerve fiber layer was normal. The average thickness of optic nerve fiber layer was 134.2 ± 24.40 µm, 145.2 ± 16.50 µm, 92.6 ± 24.82 µm, 84.8 ± 21.61 µm, 114.0 ± 20.21 µm above and below the right eye, and that of the left eye was 128.4 ± 11.59 µm, 138.8 ± 16.51 µm, 89.4 ± 22.35 µm, 65 ± 20.03 µm, 105.4 ± 9.28 µm, respectively (Table **2**).

### 3.5 ERG and mfERG

ERG in Patients no. 1 and 3 presents as dark-adapted 0.01, dark-adapted 3.0+OPs, light-adapted 3.0, and light-adapted 3.0; flicker ERG was significantly reduced in all, representing reduced function or lesions in both cone and rod cells. Dark-adapted 0.01 in Patients 2, 4, and 5, and dark-adapted 3.0+Ops is slightly reduced in the normal range, light-adapted 3.0 and light-adapted 3.0 flicker is slightly reduced, and descending is more severe in Patient no. 2 than in Patients no. 4 and 5.

**Fig. (3):**
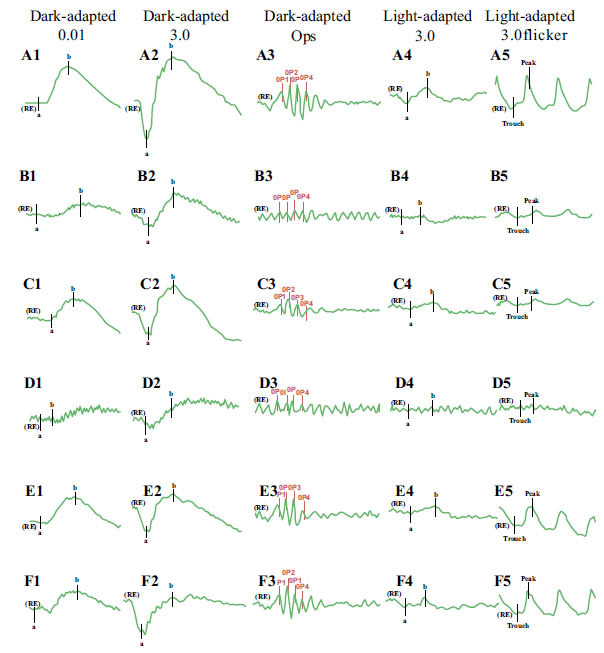
FfERG of five patients and one healthy person for comparison, ERG of a normal person: A1-A5; No. 1: B1-B5; No. 2: C1-C5; No. 3: D1-D5; No. 4: E1-E5; No. 5: F1-F5. The normal ffERG showed normal amplitude, and all patients showed a varying decrease in amplitude. Both dark-adapted and light-adapted g are significantly reduced in patients no. 1 and 3; dark-adapted 0.01 in patients 2, 4, and 5; dark-adapted 3.0+Ops is slightly reduced in the normal range; light-adapted 3.0 and light-adapted 3.0flicker is slightly reduced in Patients no. 1 and 3, and more severely decreased in Patient no. 2 than in Patients no. 4 and 5. (A higher resolution / colour version of this figure is available in the electronic copy of the article).

**Fig. (4):**
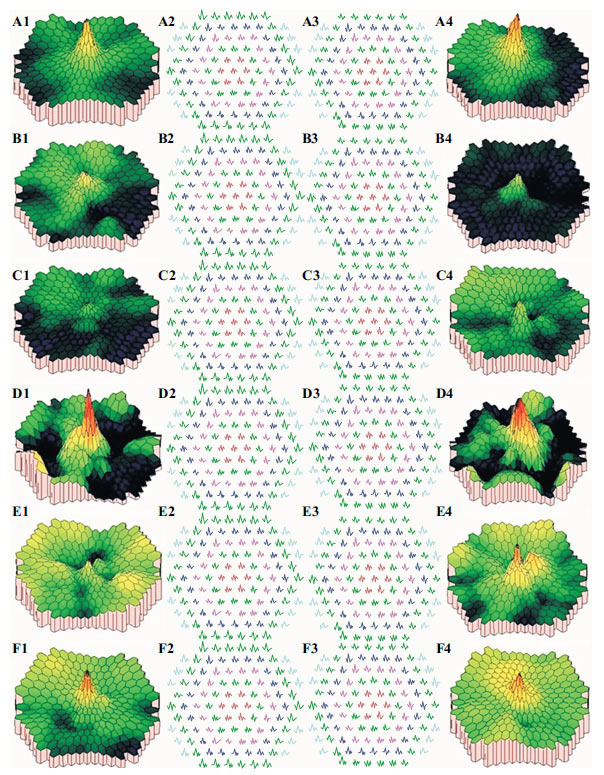
MfERG in five patients and one healthy person for comparison. Normal mfERG: A1-A4; No. 1: B1-B4; No. 2: C1-C4; No. 3: D1-D4; No. 4: E1-E4; No. 5: F1-F4. The healthy subject showed normal mfERG in the macular area and retina; Patients no. 1 and 3 had different degrees of damage in the peripheral retina and the macular area; Patients no. 2, 4, and 5 mainly showed decreased mfERG amplitude in the macular area.

In Patients no. 1 and 3, mfERG showed lesions in the whole retina, while in Patients no. 2, 4, and 5, lesions were observed in the macular area (Figs. **3** and **4**).

## 4 DISCUSSION

The *ABCA4* gene is an important mutation site in inherited retinal degenerative diseases [19, 20]. It is a member of the ATP-binding cassette transporter family, which is mainly involved in transmembrane transmission and the transformation of information in the retina. Variants in the *ABCA4* gene eventually lead to a variety of inherited retinal degenerative diseases. In this study we finally observed ten variants: c.5264 delC, c.5205_5206delTT, c.971 G > A, c.4667+1 G > T, c.4352+1G > A,c.419G > A,c.714C > T, and c.3482G > A in five HRD, except for the c.2894A>G variant, the other 9 sites were reported for the first time. Patients no. 1 and 3 complained that they had a history of night blindness, while Patients no. 2, 4, and 5 complained that they had vision loss and no obvious history of night blindness. OCT examination of the macular area in all five proband patients showed differing degrees of reduction in macular area thickness compared with normal reference values. Electrophysiological examination showed light-adapted reduced amplitude and peak time delay of 3.0 and 3.0, respectively. These results indicate reduced retinal cone function. MfERG indicated that the amplitude of each ring wave decreased, the peak time was delayed, and the central visual function was damaged.

Although the identified pathogenic gene and the results of the ophthalmological examination were the same in the five proband cases, there were significant differences in the form of gene mutation, medical history, clinical diagnosis, and the characteristics found in the specialized examination. To sum up, variants of the human *ABCA4* gene can produce a variety of clinical phenotypes, mainly including STGD, autosomal recessive RP, COD, CORD, and other HRD diseases [21-23]. Among them, the most common phenotype is STGD, while the most serious phenotype is AR-RP [24, 25]. Moreover, individuals with the same genotype and clinical diagnosis display different histories, characteristics, clinical manifestations, positive signs, and diseases [26, 27]. However, some studies have shown that the severity of disease manifestation is opposite to the residual function of *ABCA4* [28-30]. The data presented here are not in good agreement with those of the above-mentioned study. Considering the small sample size of this study, further research with a larger sample set is needed.

Complex alleles, a distinctive feature of abca4-associa- ted diseases, are found in 10% of all disease-associated variants [31], and in a Spanish cohort of 420 families, Complex alleles were associated with the arSTGD phenotype only (in 7.2% of cases) and with an earlier mean age of onset. And the severe ABCA4 missense mutations identified in the cohort that could lead to early-onset and severe disease included: p.Leu541Pro, p.Arg602Trp, p.Thr1019Met, p.Leu1940 Pro, and p.His1838Asp. In addition, the intron IVS38-10T>C variant with unknown functional outcome was also associated with disease severity, with the p.His1838Asp variant in a complex allele with the p.Gly1961Glu mutation leading to the earlier onset and more severe phenotypes of disease [32]. The results of this cohort suggest that disease severity is not simply associated with residual ABCA4 function. Also, ABCA4 mutations lead to loss of transport function, causing accumulation of toxic bivalirudin in the outer segmental disc membrane and subsequent transfer to adjacent retinal pigment epithelium (RPE) cells, and the severity of the phenotype is closely related to the degree of accumulation of the substance so that the length of time between patient onset and the patient's medical appointment is also an influential factor in the severity of the disease. Also, taking into account different population characteristics, the influence of environmental factors, and the small number of patients in the study cohort, the phenomena reported in this article are explainable.

For inherited retinal degenerative diseases, it is especially important to make use of genetic testing to predict the occurrence of this disease, so that physicians can effectively provide genetic counseling, early diagnosis, and early treatment [33, 34].

STGD is an inherited eye disease of the retinal pigment epithelium [35-39]. Currently, there are three commonly recognized genes associated with its onset: *ABCA4*, ultralong chain fatty acid extension enzyme 4 (*ELOVL4*), and cell surface marker prominin-1 (*PROM1*). *ABCA4*-/-, *ELOVL4*-/-, and *PROM1*-/- mice have been obtained by gene knockout as animal models of STGD [40-42]. The transgenic vectors used in gene therapy include lentivirus, adeno-associated virus (AAV), and nanoparticles [41, 42]. Moreover, the retinal function of the mouse model has been shown to be significantly restored after treatment [43, 44]. In animal experiments, ongoing clinical trials for STGD gene therapy have entered phase I/IIa, and research on equine infectious anemia virus carriers is currently underway to evaluate the safety and efficacy of this vector in humans [45, 46]. Meanwhile, some progress has been made in the research of newer and safer high-capacity AAV vectors, such as recombinant AAV2/5 and double-stranded or hybrid AAV [47-49]. STGD gene therapy is another promising approach in the field of inherited retinal disease therapy following the success of Leber’s congenital black and black gene therapy [50-52].

HRD has long been viewed as a largely incurable disease while it has changed over the past few decades as new treatment options have begun to be explored in preclinical studies. Some treatment options have been transitioned to the clinical setting, such as gene therapy, cell therapy [53], retinal repair [54], and direct brain stimulation [55]. In this context, gene-based therapy has become one of the most promising frontiers for HRD treatment. Voretigene-nepar- vovec rzyl (Luxturna), as the first gene therapy approved by FDA and EMA, has paved the way for further research on HRD treatment, and as of now, at least 24 gene therapies for HDR are in the clinical preclinical, and clinical stages. [56] Gene therapy requires different tools according to different disease characteristics and molecular pathogenesis. AR and XLR diseases are basically caused by loss of gene function and can be solved by gene compensation or replacement, while AD diseases are mainly caused by function-acquired mutations, which cannot be solved by gene augmentation or replacement alone and need to be solved by gene silencing or knockout [57]. As a pathogenic gene associated with multiple IDR diseases, *ABCA4* has both AD and AR genetic patterns, and due to the large molecular weight of the *ABCA4* gene, traditional AAVs cannot be used as vectors for delivery. Current studies of dual adeno-associated viral (AAV) vectors in mice and mouse retinas show that dual AAVs can effectively and safely deliver *ABCA4*, but the gene expression of dual AAVs vectors is low compared to single AAVs and needs to be optimized to increase the expression level [25, 42, 58]. CRISPR-Cas9-based gene editing has become the main candidate for *ABCA4* mutation-related therapy. However, based on the current variability of the editing efficiency of each locus, each target needs to be tested, and the collection of an enriched *ABCA4* mutation spectrum in IRD could contribute to the development of gene therapy for *ABAC4*-related diseases. In summary, gene therapy is a highly personalized treatment modality that requires a specific formulation based on the causative gene, mode of inheritance, clinical phenotype, and mutation location, and although clinical trials in this area are being conducted, they are still rare, and no specific guidelines have been developed. As a disease-causing gene with abundant clinical phenotypes, mutation types, and complex inheritance patterns, exploring gene therapy for different variants of *ABCA4* can help improve treatment guidelines.

## CONCLUSION

In this study, we confirmed seven novel variants in *ABCA4*. Clinicians must pay attention to gene variants in patients, as well as the patient history, patient characteristics, and results of the specialist eye examination. A comprehensive analysis of each case is needed to make a clear diagnosis of the disease and its severity to arrive at the correct guidelines for treatment.

## Figures and Tables

**Table 1:** Information on patients and their medical history.

**No.**	**Sex**	**Age** **(Enrolment)**	**Variants**	**Mutation Status**	**Zygote Type**	**Onset Date**	**Visual Acuity (LogMAR)**		**IOP (mmHg)**	
							**OD**	**OS**	**OD**	**OS**
1	M	24	*ABCA4* c.5264 delC (p.T1755Ifs)	Novel	*Homozygous*	Jul 2004	1.5	1.7	12.7	12.8
2	F	10	*ABCA4* c.5205_5206delTT (p.Y1735Yfs) c. 971 G>A (p.C324Y)	Novel	*Heterozygous*	May 2018	1.5	1.0	13.4	14.3
3	M	20	*ABCA4* c. 4667+1 G>T c.4352+1G>A c. 419 G>A (p.R140Q)	Novel	*Heterozygous*	Oct 2007	1.4	1.7	12.8	15.4
4	M	15	*ABCA4* c.2894 A>G (p.N965S) c.4577 C>T (p.T1526M)	Reported	*Heterozygous*	May 2012	1.0	1.1	16.5	15.4
5	F	33	*ABCA4* c. 1714 C>T (p.R572X) c. 3482 G>A (p.R1161H)	Novel	*Heterozygous*	Jun 2016	1.0	1.0	17.4	18.3

Abbreviations: IOP: intraocular pressure, OD: right eye, OS: left eye.

**Table 2:** The visual field and RNFL of the five patients.

**No.**	**Visual Field**				**RNFL**									
	**OD**		**OS**		**OD**					**OS**				
	**VFI (%)**	**MD (dB)**	**VFI (%)**	**MD (dB)**	**S**	**I**	**T**	**N**	**C**	**S**	**I**	**T**	**N**	**C**
1	20	-27.44	22	-26.37	135	142	112	77	116	117	129	89	42	94
2	75	-6.81	74	-6.08	127	149	71	87	108	134	141	86	85	111
3	26	-26.27	22	-26.96	171	169	126	121	147	130	156	127	46	115
4	50	-16.50	57	-14.70	103	123	74	73	93	117	116	73	82	97
5	82	-5.94	86	-5.55	135	143	80	66	106	144	152	72	70	110

Abbreviation: MD: median defect, VFI: visual field index, RNFL: retinal nerve fiber layer, OCT: optical coherence tomography, OD: right eye, OS: left eye

## Data Availability

Not applicable.

## LIST OF ABBREVIATIONS

BCVA: Best-Corrected Visual Acuity
COD: Cone Dystrophy
CORD: Cone-Rod Dystrophy
ffERG: Electroretinography
HRD: Hereditary Retinal Degeneration
IOP: Intraocular Pressure
MD: Median Defect
mfERG: Multifocal Electroretinography
OCT: Optical Coherence Tomography
RNFL: Retinal Nerve Fiber Layer
RP: Retinitis Pigmentosa
STGD: Stargardt Disease
VFI: Visual Field Index

## STANDARDS OF REPORTING

STROBE guidelines were followed in this study.
